# ﻿On the enigmatic jumping spider genus *Ogdenia* Peckham, 1908 (Araneae, Salticidae, Chrysillini)

**DOI:** 10.3897/zookeys.1202.124199

**Published:** 2024-05-16

**Authors:** Zhiyong Yang, Quang Duy Hoang, Weihang Wang, Wayne P. Maddison, Junxia Zhang

**Affiliations:** 1 Key Laboratory of Zoological Systematics and Application, College of Life Sciences, Hebei University, Baoding, Hebei 071002, China; 2 Hebei Basic Science Center for Biotic Interaction, Hebei University, Baoding, Hebei 071002, China; 3 Faculty of Natural Sciences and Technology, Tay Nguyen University, 567 Le Duan, Buon Ma Thuot, Dak Lak 630000, Vietnam; 4 Departments of Zoology and Botany and Beaty Biodiversity Museum, University of British Columbia, 6270 University Boulevard, Vancouver, British Columbia, V6T 1Z4, Canada

**Keywords:** Chrysillini, *Ogdeniamutilla*, redescription, Southeast Asia

## Abstract

The monotypic genus *Ogdenia* Peckham, 1908, is redefined based on the redescription of the holotype of *O.mutilla* (Peckham & Peckham, 1907), along with the newly discovered male specimens and intraspecific variation from China, Malaysia, Singapore, and Vietnam. Description, illustrations, and photographs are provided.

## ﻿Introduction

In 1907, G.W. Peckham and E.G. Peckham established the monotypic genus *Rooseveltia* with the type species *Rooseveltiamutilla* Peckham & Peckham, 1907, based on a single female holotype from Borneo, Kuching, Malaysia (Peckham and Peckham 1907). *Rooseveltia* was later renamed *Ogdenia* due to its homonymy with *Rooseveltia* Jordan & Evermann, 1906 in Pisces (Peckham 1908). With the absence of diagnostic illustration in the original description, this genus long remained enigmatic until Prószyński (1984) provided drawings for the epigyne and retromarginal tooth based on the holotype of the type species. In addition, photographs of living females of *O.mutilla* from Singapore and Malaysia (Johor and Sarawak) were recently provided in "*A Photographic Guide to Singapore Spiders*" (Joseph et al. 2022). Although the information about *Ogdenia* has been accumulated in past decades, the male of this genus remained unknown.

Herein, we redescribe *O.mutilla* and provide photographs of the habitus and epigyne of the holotype specimen. In addition, we document the male for the first time. Furthermore, intraspecific variation based on specimens from China, Malaysia, Singapore, and Vietnam is presented.

## ﻿Materials and methods

Specimens preserved in 75% or 95% ethanol were examined and measured under a Leica M205A stereomicroscope. All measurements are in millimetres using the associated Leica LAS v. 4.3 software. Photographs of living specimens were captured using a Canon 80D camera equipped with a Laowa 100 mm f/2.8 macro 2× lens and a KR-888 flash, a Sony ILCE-7RM2 camera with a Laowa 90 mm f/2.8 macro 2× lens and a KR-808 flash, and a Nikon D7200 camera with an AF-S DX Macro Nikkor 40 mm f/2.8G lens and a KR-888 flash. Ethanol-immersed body and genitalia photos were taken by a Kuy Nice CCD camera mounted on an Olympus BX53 microscope and then stacked by the Helicon Focus v. 7 software. Final photographs were retouched in Adobe Photoshop CC 2021. The male palp was macerated in clove oil to observe the trajectory of the spermophor, while the female vulva was cleaned with pancreatin. The holotype specimen is preserved in the Museum of Comparative Zoology, Harvard University, USA (**MCZ**); the examined specimens from China are preserved in the Museum of Hebei University, Baoding, China (**MHBU**), those from Vietnam are preserved at the Tay Nguyen University, Buon Ma Thuot, Vietnam (**TNU**), and those from Singapore and Malaysia are preserved in the Beaty Biodiversity Museum, University of British Columbia, Vancouver, Canada (**UBCZ**).

Abbreviations used: **AG**, accessory gland; **ALE**, anterior lateral eye; **AME**, anterior median eye; **BH**, basal haematodocha; **C**, cymbium; **CD**, copulatory duct; **CO**, copulatory opening; **E**, embolus; **FD**, fertilization duct; **IZ**, Invertebrate Zoology; **PL**, posterior tegular lobe; **PLE**, posterior lateral eye; **PME**, posterior median eye; **RTA**, retrolateral tibial apophysis; **S**, spermatheca; **SM**, spermophor; **TB**, tegular bump; **XTBG**, Xishuangbanna Tropical Botanical Garden.

## ﻿Taxonomy

### ﻿Genus Ogdenia

Taxon classificationAnimaliaAraneaeSalticidae

Peckham, 1908

6B5105E7-5AD5-59C7-A880-C1486459CB67

Rooseveltia Peckham & Peckham, 1907: 614; type species: Rooseveltiamutilla Peckham & Peckham, 1907, by original designation and monotypy.
Ogdenia Peckham, 1908: 171 (generic replacement name).

#### Diagnosis.

**Figure 1.**
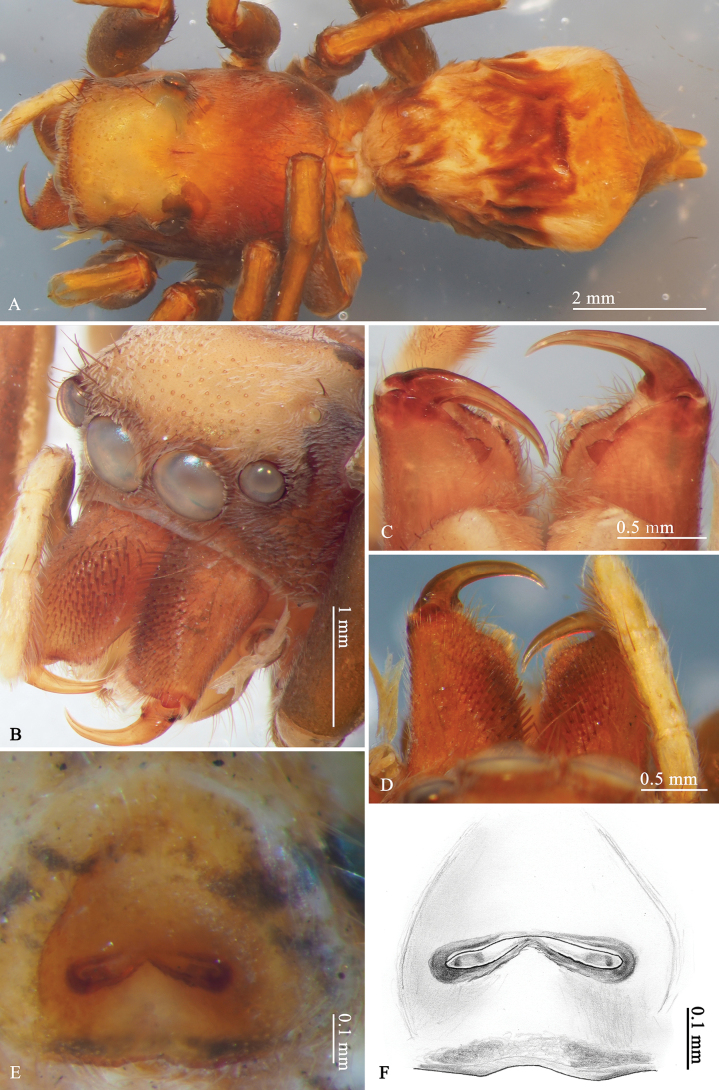
*Ogdeniamutilla* (Peckham & Peckham, 1907), female holotype (©2024 W.P. Maddison) **A** habitus, dorsal view **B** prosoma **C, D** chelicerae, front (**D**) and back (**C**) views **E, F** epigyne, ventral views.

The genus closely resembles *Siler* Simon, 1889, but can be distinguished by: (1) the larger body size (6–10 mm); (2) the male femur I with only sparse setae (Figs 4B, E, F, 5O; femur I and tibia I covered with dense setae in *Siler*); (3) the male palp with a blunt posterior tegular lobe (PL) (Figs 8B, C, 10A, 11E, 12D; PL longer and narrower in *Siler*); (4) the chelicerae with dense brown setae on the front surface (Figs 1B, D, 5M, N, P, Q, T, U, 6E, F, 7A, C, E; setae sparse in *Siler*); (5) the presence of accessory glands (AGs) (Figs 9B–F, 10D, 11B, D; AG not observed in *Siler*).

#### Description.

Medium-sized spiders (total length 6.51–8.00 in males, 7.98–10.00 in females). Body dark with pale yellow patches, covered with dense scales (Figs 3A, B, 4A–L, 5A–D, 6A–D, 7B, D, F). Chelicerae with brown setae on front surface (male also with sparse blue scales), with two promarginal teeth and one retromarginal tooth (Figs 1C, D, 3C, 4B, E, F, H, 5P–U). Femur I of male covered with short and sparse setae (Figs 4B, E, F, 5O). Male palp (Figs 8A–F, 10A, B, 11E, 12C–E) with short embolus, palpal bulb with tegular bump at lower right corner. Female epigyne large, copulatory ducts short (Figs 9A–G, 10C, D, 11A–D, 12A, B).

#### Distribution.

China (Yunnan), Malaysia (Borneo, Johor, and Sarawak), Singapore (Bukit Timah Nature Reserve), Vietnam (Dak Lak).

### ﻿ Ogdenia mutilla

Taxon classificationAnimaliaAraneaeSalticidae

(Peckham & Peckham, 1907)

F545DC8E-AB61-5C1B-B9A7-D3C2C8BEF137

Figs 1 , 2 , 3 , 4 , 5 , 6 , 7 , 8 , 9 , 10 , 11 , 12 缪恐翠蛛

Rooseveltia mutilla Peckham & Peckham, 1907: 614; Prószyński 1984: 125.
Ogdenia mutilla : Peckham, 1908: 171; Prószyński 2017: 129, fig. 56I.

#### Type material.

***Holotype***: Malaysia • ♀; Borneo, Kuching; 1 Jan. 1897; R. Shelford & Peckham leg.; MCZ: IZ: 22236, examined.

#### Other material examined.

China – Yunnan Province • 7♂♂ 2♀♀; Xishuangbanna Dai Autonomous Prefecture, Mengla County, Menglun Town, XTBG; 21.9080°N, 101.2845°E, 669 m a.s.l.; 21 Apr. 2023; W. Wang & Z. Yang leg.; MHBU-ARA-00025193, 00025196, 00026514–00026517 • 1♀; same collection data as for preceding; 21.9077°N, 101.2824°E, 677 m a.s.l.; 17 Dec. 2022; W. Wang, B. Liu & Z. Yang leg.; MHBU-ARA-00025194 • 2♀♀; same collection data as for preceding; 21.9108°N, 101.2833°E, 669 m a.s.l.; 2 Aug. 2021; J. Zhang, Y. Mu, K. Yu, L. Zhang & W. Wang leg.; MHBU-ARA-00022704, 00022804 • 1♀; same collection data as for preceding; 21.9092°N, 101.2805°E, 605 m a.s.l.; 12 Jul. 2018; C. Jin & C. Zhang leg.; MHBU-ARA-00020807 • 1♂; Xishuangbanna Dai Autonomous Prefecture, Mengla County, Wangtianshu scenic area; 21.6223°N, 101.5892°E, 700 m a.s.l.; 23 Apr. 2023; L. Wang & Q. Lu leg.; MHBU-ARA-00025195.

Malaysia – 1♂; Johor, Gunung Belemut Forest, Lata Tengkorak; 2.0550°N, 103.5430°E, 250 m a.s.l.;16 Jun. 2019; W.P. Maddison, N.I. Morehouse et al. leg.; UBCZ.

Singapore – 1♀; Bukit Timah Nature Reserve, South Jungle Falls Path; 1.3551°N, 103.7739°E, 150 m a.s.l.; 5 Jun. 2019; W.P. Maddison leg.; UBCZ • 1♀; same collection data as for preceding; 1.3550°N, 103.7740°E–1.3570°N, 103.7750°E, 150–160 m a.s.l.; 12–14 Jun. 2019; W.P. Maddison, N.I. Morehouse et al. leg.; UBCZ.

Vietnam – Dak Lak Province • 1♂; Buon Don District, Yok Don National Park; 12.8641°N, 107.7961°E, 180 m a.s.l.; 24 Apr. 2022; Q.D. Hoang leg.; TNU • 1♀ Krong Bong District, Chu Yang Sin National Park; 12.4796°N, 108.3391°E, 430 m a.s.l.; 14 May 2022; Q.D. Hoang leg.; TNU.

#### Diagnosis.

See the diagnosis of the genus.

#### Description.

**Male.** Habitus as shown in Figs 3A–C, 4A–G, L, 5A, B, 6C, D, 7F). Measurements (MHBU-ARA-00025193): carapace 3.27 long, 2.65 wide, abdomen 4.19 long, 2.39 wide; measurements of eyes: AME 0.55, ALE 0.31, PME 0.09, PLE 0.34; measurements of legs: I 10.52 (3.42, 1.30, 2.91, 1.80, 1.09), II 6.92 (2.20, 0.97, 1.67, 1.19, 0.89), III 7.54 (2.18, 0.90, 1.60, 1.72, 1.14), IV 10.07 (3.07, 1.03, 2.36, 2.47, 1.14); leg formula 1432. Body black and pale yellow except for blue carapace edge, covered with dense scales (Figs 3A–C, 4A–G, L, 5A, B, 6C, D, 7F). Chelicerae claybank color, with sparse blue scales (alive) and relatively dense setae on front surface, with two promarginal and one retromarginal tooth (Figs 5M, P–R, 6F, 7E). Femur I covered with short setae (Figs 4B, E, F, 5O). Number of teeth in each tarsal claw varied (Fig. 5E–L).

**Palp** (Figs 8A–F, 10A, B, 11E, 12C–E): embolus short; cymbium yellow, longer than wide; retrolateral tibia apophysis around one-third of bulb length; palpal bulb like wax apple, with tegular bump at lower right corner.

**Female.** Habitus as shown in Figs 1A, 4H–K, 5C, D, 6A, B, 7B, D. Measurements (MHBU-ARA-00025194): carapace 3.45 long, 2.81 wide; abdomen 4.70 long, 3.04 wide; measurements of eyes: AME 0.58, ALE 0.35, PME 0.10, PLE 0.30; measurements of legs: I 8.11 (2.54, 0.96, 2.15, 1.47, 0.99), II 6.72 (2.18, 0.84, 1.62, 1.20, 0.88), III 7.71 (2.27, 0.94, 1.67, 1.87, 0.96), IV 10.5 (3.15, 0.91, 2.52, 2.87, 1.05); leg formula 4132. Body form and color pattern same as male, but without short setae on femur I. Chelicerae lacking blue scales, with slightly different shapes of teeth from males (Figs 1C, D, 4H, 5S–U, 6E, 7A, C).

**Figure 2.**
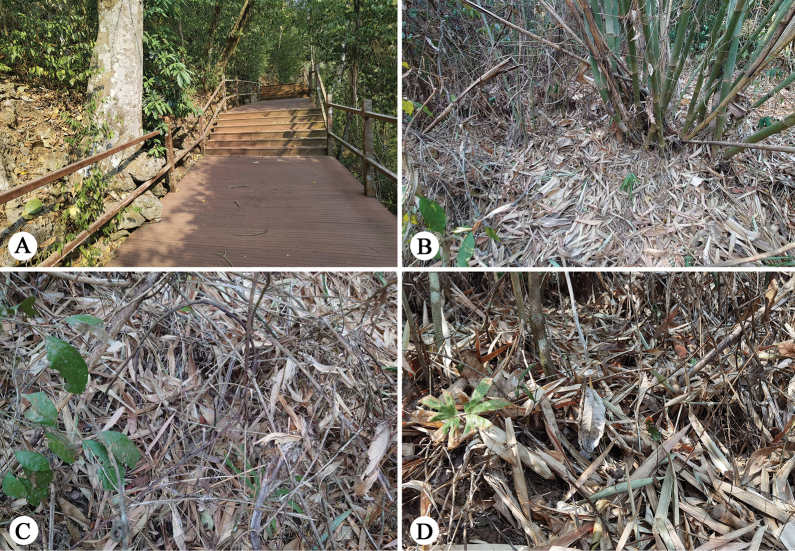
Habitats of *Ogdeniamutilla* (Peckham & Peckham, 1907) in XTBG, Yunnan, China.

**Figure 3.**
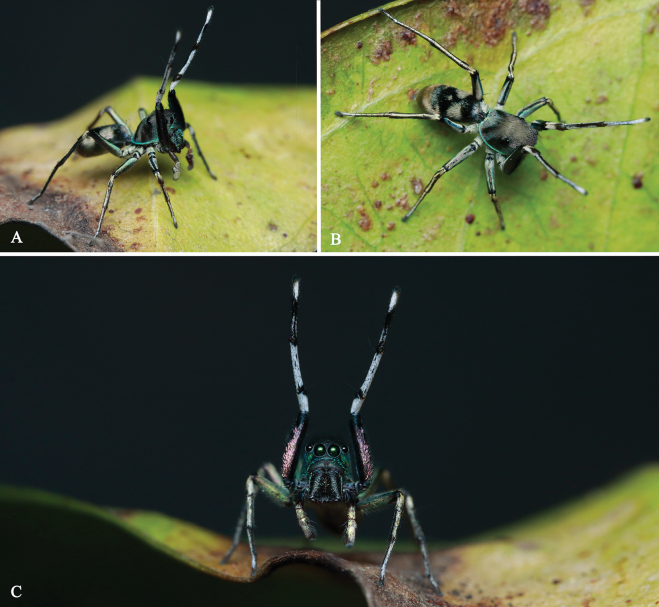
Living males of *Ogdeniamutilla* (Peckham & Peckham, 1907) from China (©2023 Q. Lu).

**Figure 4.**
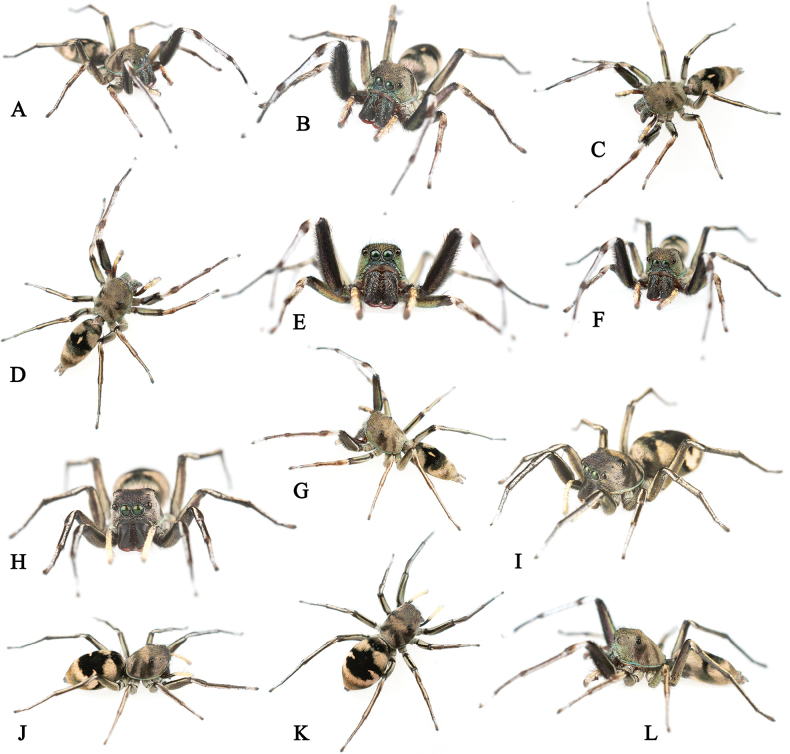
*Ogdeniamutilla* (Peckham & Peckham, 1907) from China **A–G, L** living males **H–K** living females.

**Figure 5.**
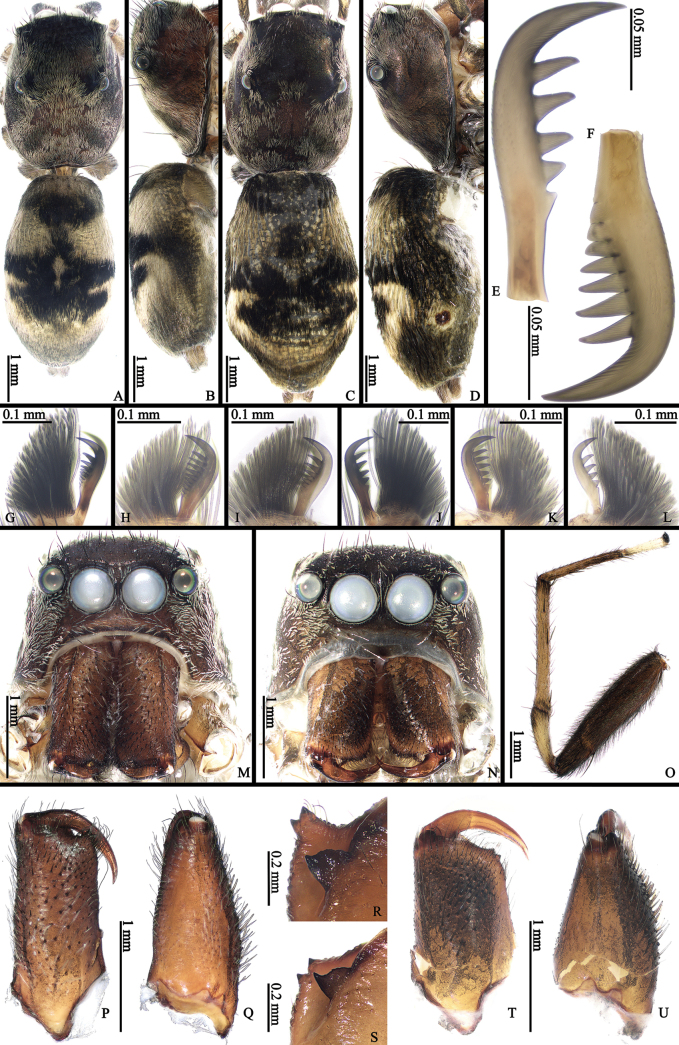
*Ogdeniamutilla* (Peckham & Peckham, 1907), male (**A, B, E–M, O–R**) and female (**C, D, N, S–U**) from China **A–D** habitus, dorsal (**A, C**) and lateral (**B, D**) views **E–L** claws I (**G, J**), II (**H, K**), III (**I, L**), IV (**E, F**), prolateral (**F–I**) and retrolateral (**E, J–L**) views **M, N** prosomas **O** left leg I, front view **P, Q, T, U** chelicerae, front (**P, T**) and prolateral (**Q, U**) views **R, S** teeth of chelicerae, back views.

**Figure 6.**
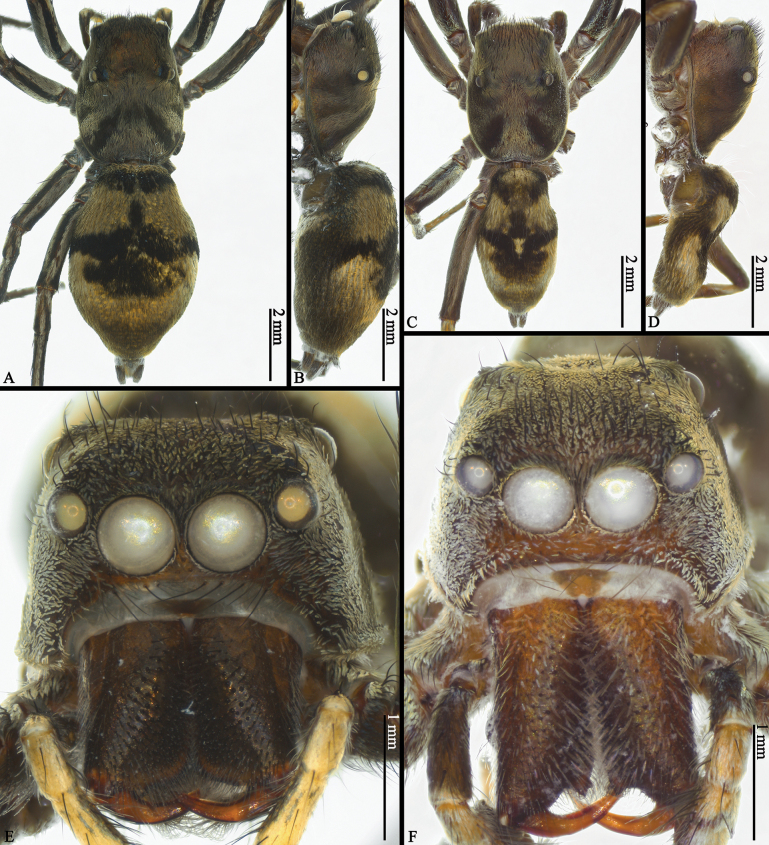
*Ogdeniamutilla* (Peckham & Peckham, 1907), male (**C, D, F**) and female (**A, B, E**) from Vietnam (©2023 Q.D. Hoang) **A–D** habitus, dorsal (**A, C**) and lateral (**B, D**) views **E, F** prosomas.

**Figure 7.**
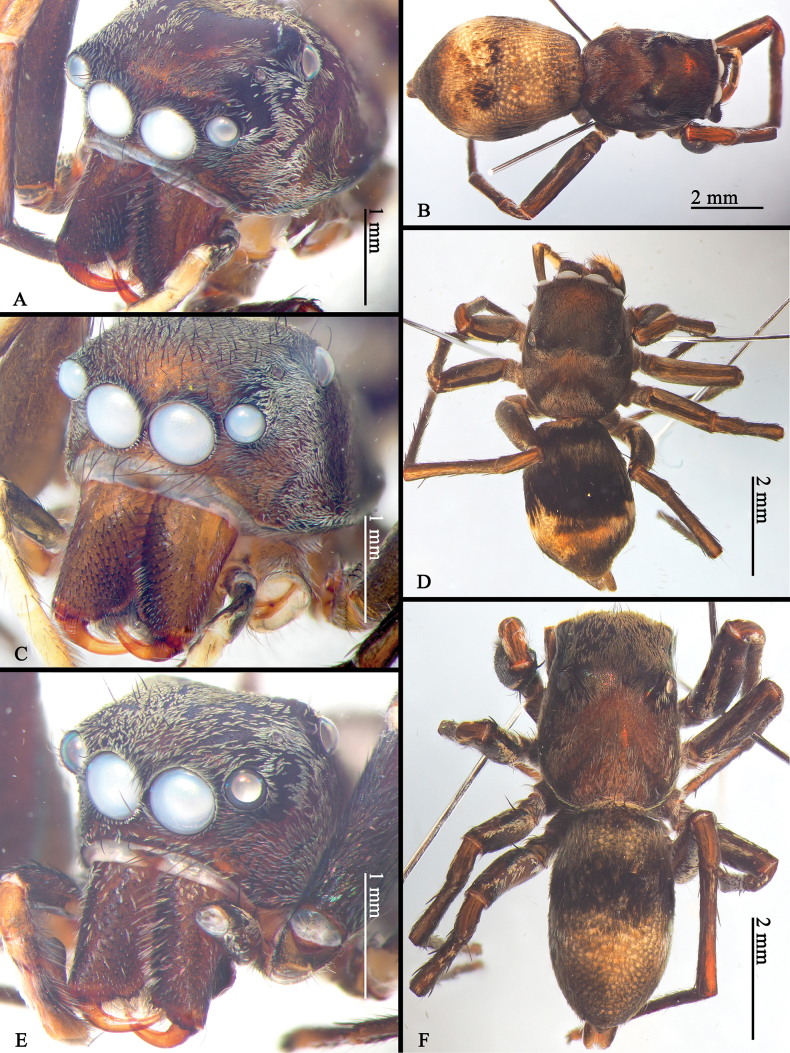
*Ogdeniamutilla* (Peckham & Peckham, 1907), male (**E, F**) and female (**A–D**) from Singapore (**A–D**) and Malaysia (**E, F**) (©2024 W.P. Maddison) **A, C, E** prosomas **B, D, F** habitus, dorsal views.

**Epigyne** (Figs 1E, F, 9A–G, 10C, D, 11A–D, 12A, B): copulatory openings elongate like curved butterfly antennae; copulatory ducts short; fertilization ducts at anterior of spermathecae; accessory glands small, located close to the junction between spermathecae and copulatory ducts.

**Figure 8.**
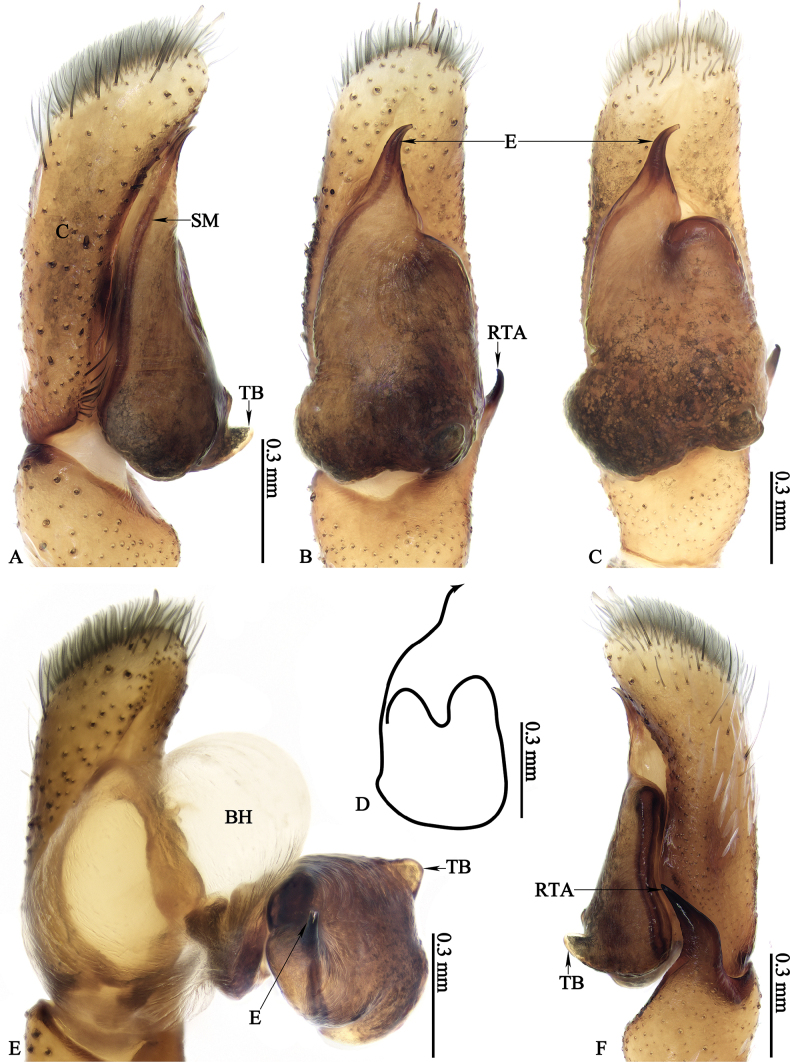
*Ogdeniamutilla* (Peckham & Peckham, 1907) from China **A–C, E, F** male left palp, prolateral (**A**), ventral (**B, C**), pro-ventral (**E**) and retrolateral (**F**) views **D** spermophor, ventral view **B, C** showing the intraspecific variation of the male palp.

**Figure 9.**
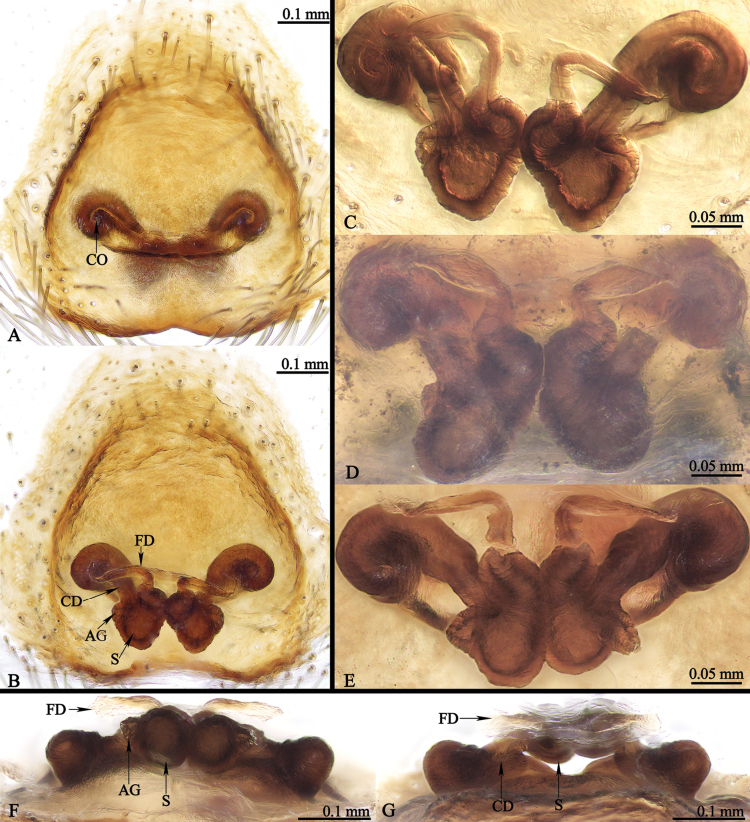
*Ogdeniamutilla* (Peckham & Peckham, 1907) from China. **A** epigyne, ventral view **B–G** vulvae, dorsal (**B–E**), front (**G**) and back (**F**) views **C–E** showing the intraspecific variation of the detailed structures of the vulvae.

**Figure 10.**
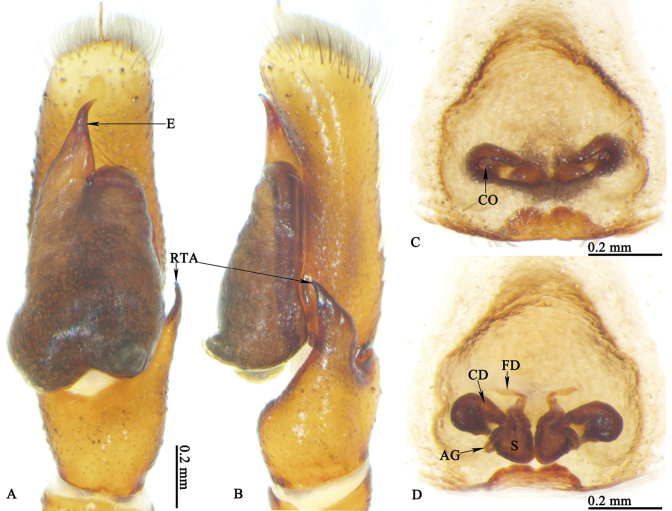
*Ogdeniamutilla* (Peckham & Peckham, 1907) from Vietnam (©2023 Q.D. Hoang) **A, B** male left palp, ventral (**A**) and retrolateral (**B**) views **C** epigyne, ventral view **D** vulva, dorsal view.

**Figure 11.**
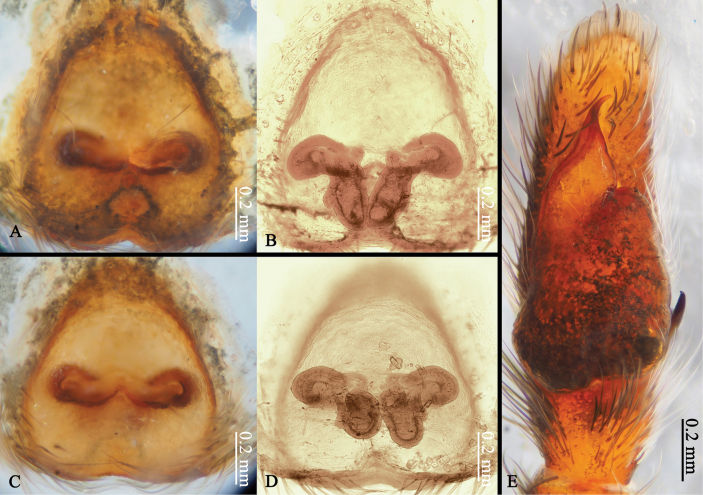
*Ogdeniamutilla* (Peckham & Peckham, 1907) from Singapore (**A–D**) and Malaysia (**E**) (©2024 W.P. Maddison) **A, C** epigynes, ventral views **B, D** vulvae, dorsal views **E** male left palp, ventral view.

**Figure 12.**
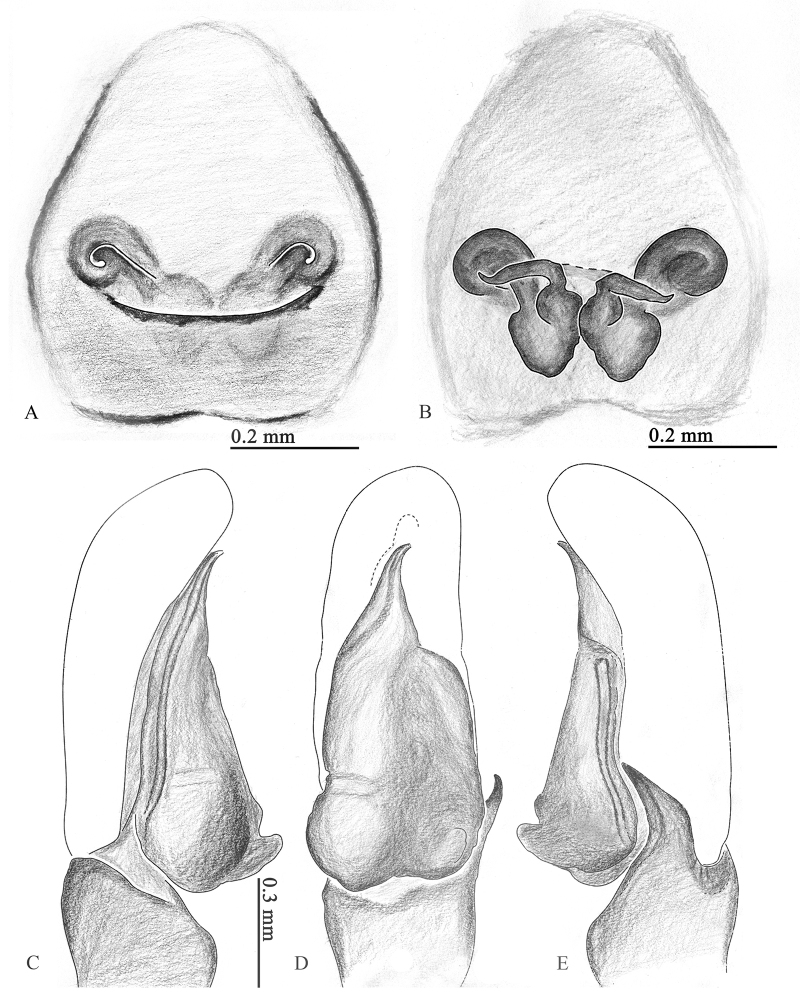
*Ogdeniamutilla* (Peckham & Peckham, 1907) from China **A** epigyne, ventral view **B** vulva, dorsal view **C–E** male left palp, prolateral (**C**), ventral (**D**) and retrolateral (**E**) views.

#### Variation.

The transition from the base of the embolus to the tegulum is smoother in some specimens (Figs 8B, 11E), but has an obvious junction in others (Figs 8C, 10A). For the females, differences in the size of accessory glands, the shape of spermathecae, copulatory openings and middle rim of epigyne are observed among different individuals (Figs 1E, F, 9A–E, 10C, D, 11A–D, 12A, B).

#### Natural history.

Specimens were discovered on the surface of leaf litter or in shrubs within tropical forests (Fig. 2A–D), moving actively and quickly. These specimens were collected during relatively dry seasons, and the subsequent lab observations showed that they may not prefer highly humid environments. While the original description of *O.mutilla* suggested that the species mimics *Mutilla* Linnaeus, 1758, a genus of parasitoid wasps (Peckham and Peckham 1907), our field observations indicated that *O.mutilla* possibly mimics large sympatric ants, similar to the imperfect ant-mimicry phenomenon reported in some species of *Siler* (Zeng et al. 2023).

#### Distribution.

China, Malaysia, Singapore, and Vietnam.

## Supplementary Material

XML Treatment for Ogdenia

XML Treatment for Ogdenia mutilla

## ﻿Additional information

### Author ORCIDs

Zhiyong Yang https://orcid.org/0000-0002-7610-6843

Quang Duy Hoang https://orcid.org/0000-0003-1672-7960

Weihang Wang https://orcid.org/0000-0003-2460-4514

Wayne P. Maddison https://orcid.org/0000-0003-4953-4575

Junxia Zhang https://orcid.org/0000-0003-2179-3954

### Data availability

All of the data that support the findings of this study are available in the main text.

## References

[B1] Joseph KHK David JC Chris SPA Paul YCN (2022) A photographic guide to Singapore spiders. Singapore Botanic Gardens, Singapore, 774 pp.

[B2] Linnaeus C (1758) Systema naturae per regna tria naturae, secundum classes, ordines, genera, species, cum characteribus, differentiis, synonymis, locis. Tomus I. Editio Decima Reformata. Laurentii Salvii, Holmiae, Stockholm, 823 pp. 10.5962/bhl.title.542

[B3] Peckham GW (1908) The generic name *Rooseveltia.* Bulletin of the Wisconsin Natural History Society 6: 171.

[B4] Peckham GW Peckham EG (1907) The Attidae of Borneo. Transactions of the Wisconsin Academy of Sciences, Arts, and Letters 15(2): 603–653.

[B5] Prószyński J (1984) Atlas rysunków diagnostycznych mniej znanych Salticidae (Araneae). Zeszyty Naukowe Wyższej Szkoły Rolniczo-Pedagogicznej w Siedlcach 2: 1–177.

[B6] Prószyński J (2017) Pragmatic classification of the world’s Salticidae (Araneae). Ecologica Montenegrina 12: 1–133. 10.37828/em.2017.12.1

[B7] Zeng H Zhao D Zhang Z Gao H Zhang W (2023) Imperfect ant mimicry contributes to local adaptation in a jumping spider. iScience 26(6): 106747. 10.1016/j.isci.2023.106747 37378345 PMC10291251

